# EXPLORATION OF CARE CONTENTS AFFECTING STRESS VARIABILITY IN FAMILY CAREGIVERS OF OLDER PEOPLE WITH DEMENTIA

**DOI:** 10.1093/geroni/igac059.2809

**Published:** 2022-12-20

**Authors:** Sonoko Kabaya, Chieko Greiner, Masahide Nakamura, Yuko Yamaguchi, Zhang Xuanrong

**Affiliations:** Kobe University, Kobe City, Hyogo, Japan; Kobe University, Kobe City, Hyogo, Japan; Kobe University, Kobe City, Hyogo, Japan; Kobe University, Kobe City, Hyogo, Japan; Kobe University, Kobe City, Hyogo, Japan

## Abstract

Support for family caregivers (FCs) caring for older people with dementia in the home settings is a common issue in developed countries. Previous studies have reported that the increase in the burden of FCs not only causes health problems, but also makes it difficult to continue care at home and causes serious problems such as abuse. However, it is not clear how FCs’ stress, which leads to increased burden, fluctuates within a day through daily care activities. The aim of this study was to explore care activities that influence the increase in FCs’ stress. We recruited one dyad of an older adult with dementia over the age of 65 and a FC. Data collection was performed for consecutive 7 days throughout 24 hours. We adopted hamon® developed by Mitsufuji Corp. Japan to measure Heart Rate Variability (HRV)-based stress. Moreover, we asked a FC to talk with Virtual Agent on a laptop freely to understand when and what kind of care activities were done and how FC’s feelings were. Kruskal-Wallis’s test and Bonferroni’s multiple comparison test were used to compare differences in stress in the care contents. Results showed that sleeping care, which is composed of such as assistance in using the toilet and changing clothes, gives more stress to FC, compared with morning care which includes such as assistance in changing clothes, eating breakfast, and taking medicine (p=0.042). this finding is beneficial for considering how to reduce FC’s stress on daily care activities.

